# Low‐dose cannabidiol increases plasma concentrations of amitriptyline: A clinical drug–drug interaction study

**DOI:** 10.1002/bcp.70415

**Published:** 2025-12-15

**Authors:** Andriy A. Gorbenko, Titiaan E. Post, Pamela K. Strugala, Erica S. Klaassen, Linda E. Klumpers, Saco J. de Visser, Cristina Sempio, Jost Klawitter, Jules A. A. C. Heuberger, Geert J. Groeneveld

**Affiliations:** ^1^ Centre for Human Drug Research Leiden The Netherlands; ^2^ Leiden University Medical Centre Leiden The Netherlands; ^3^ Verdient Science LLC. Denver CO USA; ^4^ Tomori Pharmacology Inc. Denver CO USA; ^5^ Larner College of Medicine University of Vermont Burlington VT USA; ^6^ Centre for Future Affordable and Sustainable Therapy development (FAST) the Hague the Netherlands; ^7^ Department of Anesthesiology University of Colorado Aurora Colorado USA

**Keywords:** amitriptyline, cannabidiol, CBD, CYP2C19, DDI, interaction, tramadol

## Abstract

**Aims:**

Cannabidiol (CBD), the main non‐intoxicating compound from the cannabis plant, is regularly used by patients with chronic pain who also take analgesics. CBD has previously been shown to inhibit CYP‐mediated drug metabolism. This study aimed to characterize the potential pharmacokinetic interaction of CBD with amitriptyline and tramadol, two commonly used analgesics.

**Methods:**

This was an open‐label, fixed‐sequence, 2‐way crossover study in 13 healthy participants. On Day 1, 25 mg amitriptyline and 50 mg tramadol were co‐administered orally in a fasted condition, followed by a 7‐day washout period. On Day 8, 30 mg CBD was administered orally 1 h prior to amitriptyline and tramadol. Pharmacokinetic sampling was performed for CBD, amitriptyline, tramadol and their respective active metabolites nortriptyline and O‐desmethyltramadol for up to 24 h post‐dose. The areas under the curve (AUCs) were compared between visits using a mixed effects model.

**Results:**

Twelve participants (4M/8F) completed the study; one participant (M) dropped out for personal reasons. CBD significantly increased the AUC_0‐24h_ (least square means [LSM] ratio 1.13, 95% CI: 1.01, 1.26, **p = 0.033)** and the C_max_ (LSM ratio 1.17, 95% CI: 1.01, 1.36, **p = 0.041**) of amitriptyline. CBD did not significantly change the AUC_0‐24h_ and C_max_ of nortriptyline and tramadol, and the C_max_ of O‐desmethyltramadol.

**Conclusions:**

A single dose of 30 mg CBD significantly influenced the metabolism of amitriptyline in healthy volunteers. In patients, CBD‐induced drug interactions may be more pronounced in chronic dosing and dependent upon prandial status.

**What is already known about this subject**

- Cannabidiol (CBD) is a CYP enzyme inhibitor known to increase plasma concentrations of THC, anti‐epileptics and other drugs.
- Amitryptiline and tramadol are commonly used for the treatment of chronic neuropathic pain.
- As CYP substrates, amitryptiline and tramadol are potentially liable to drug interactions with CBD.

**What this study adds**

- Single doses of 30 mg CBD significantly increased plasma concentrations of amitriptyline in healthy volunteers.
- Concentrations of nortriptyline, tramadol and O‐desmethyltramadol did not increase significantly.
- Even a relatively low dose of CBD can influence the metabolism of drugs used by patients with chronic pain, leading to higher plasma concentrations.

## 1 INTRODUCTION

Cannabidiol (CBD) is the main non‐intoxicating compound from the cannabis plant.^1^ CBD is commonly used in combination with traditional analgesics by virtue of its frequent presence in medicinal cannabis, which is a moderately effective treatment for neuropathic pain.^2^ Moreover, CBD‐containing products such as oils, gummies and beverages are available as over‐the‐counter (OTC) health supplements^3^ and are increasingly used for treatment of pain, as well as anxiety, and improvement of sleep and mood,^4^ despite lacking clear evidence of therapeutic efficacy.^5^

Although CBD use is highly prevalent,^6^ it may not be harmless. Besides the risk of liver injury after long‐term use,^7^ CBD is also a potent CYP‐enzyme inhibitor, having been shown to inhibit mostly CYP2C19‐, CYP2C9‐, CYP3A4‐, CYP1A2‐ and CYP2B6‐mediated metabolism in vitro,^8^, ^9^, ^10^, ^11^ as well as CYP2C19, CYP2C9, CYP3A4 and CYP1A2 in humans administered a validated CYP substrate cocktail.^12^ Although some in‐vitro studies suggest a degree of CYP2D6 inhibition,^8^, ^9^, ^13^ the only available study in humans failed to show it.^12^ Clinical publications report interactions between CBD and tacrolimus,^14^ clobazam^15^ and other anti‐epileptics.^16^ Typically, the interactions between CBD and substrate drugs result in increased substrate concentrations and therefore increased toxicity, although in the case of clobazam, in has even been argued that the interaction could explain the *efficacy* of CBD in the treatment of Dravet syndrome,^17^ and the European Medicines Agency (EMA) approved CBD for this indication only in conjunction with clobazam.^18^

**FIGURE 1:**
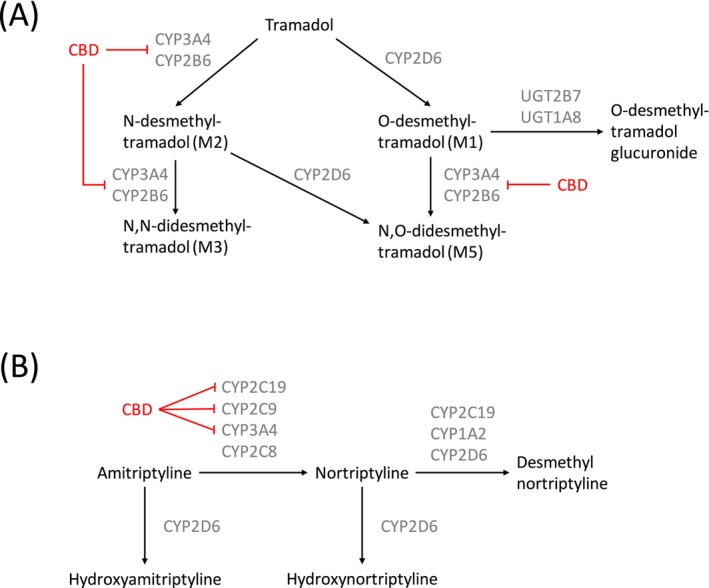
Expected action of CBD on tramadol and amitriptyline metabolism.

Patients with chronic neuropathic pain who use medicinal cannabis or OTC CBD supplements are, therefore, at risk of drug interactions between CBD and conventional analgesics that are metabolized by CYP‐enzymes. Amitriptyline is one such commonly prescribed analgesic that is liable to a pharmacokinetic interaction with CBD, as it is metabolized by CYP2C19 (mainly), CYP2C9 and CYP3A4, into nortriptyline, an active metabolite. CBD is a known inhibitor of these three enzymes and can be expected to inhibit the conversion of amitriptyline to nortriptyline, resulting in elevated amitriptyline concentrations and an increased amitriptyline to nortriptyline ratio (Figure 1). This is associated with an increased potential for adverse effects, including sedation, orthostatic hypotension and cardiotoxicity.^19^

Tramadol is another analgesic commonly used by patients with chronic neuropathic pain that has potential for drug interactions with CBD. Tramadol is converted for approximately 80% into O‐desmethyltramadol (O‐DSMT) by CYP2D6, and for approximately 20% into N‐desmethyltramadol (N‐DSMT) by CYP3A4 and CYP2B6. O‐DSMT has a significantly higher affinity for the μ‐opioid receptor than the parent compound and is primarily responsible for the analgesic effects of tramadol, whereas N‐DSMT is inactive.^20^ O‐DSMT subsequently undergoes phase II metabolism, either directly or after conversion to the mildly active N,O‐desmethyltramadol (N,O‐DSMT), by CYP3A4 and CYP2B6. CBD is an inhibitor of CYP3A4 and CYP2B6, and therefore, we hypothesized that CBD would 1) further increase the proportion of parent compound converted to O‐DSMT by CYP2D6 (which we assumed to be unaffected by CBD^12^) and 2) inhibit the N‐demethylation of O‐DSMT to N,O‐DSMT, with both 1) and 2) resulting in increased concentrations of O‐DSMT (Figure 1). Elevated O‐DSMT levels can cause toxicity such as sleepiness, confusion, shallow breathing and even life‐threatening respiratory depression.^21^

Although previous clinical research clearly demonstrated CBD‐induced CYP‐mediated drug interactions, the high doses of CBD administered limit the translatability to patients with neuropathic pain and OTC CBD users. Some studies administered a single dose of 640 mg CBD,^12^, ^22^ while others involved the long‐term treatment of rare epilepsy syndromes, with daily doses up to 50 mg/kg CBD.^14^, ^15^, ^16^ In contrast, patients with chronic neuropathic pain who participated in a clinical trial of Sativex®, a formulation containing both THC and CBD in an (approximately) equal measure, self‐administered an average of 27.3 mg CBD daily.^23^ It is likely that this lower CBD intake is also more representative of OTC CBD use, with a typical strength per serving ranging between 5 and 50 mg CBD,^3^ although products up to 100 mg CBD per serving are available.^24^ Our own previous research showed that 30 mg CBD affects the metabolism of THC (CYP3A4 and CYP2C9 substrate), demonstrating that commonly used low CBD doses may already put individuals at risk of drug interactions.^25^

The extent of pharmacokinetic interactions between CBD in commonly used doses and analgesics is not yet known. This lack of knowledge puts patients with chronic neuropathic pain, as well as other patient populations commonly treated with amitriptyline and tramadol, at an increased risk of adverse effects. The aim of this study was to characterize the potential of CBD to induce CYP‐mediated drug interactions with amitriptyline and tramadol. We used an open‐label, fixed sequence two‐way crossover design in healthy volunteers, utilizing a CBD dose that is representative of common use in patients with neuropathic pain and OTC CBD users.

## 2 METHODS

### 2.1 Participants and study design

This study was an open‐label, fixed sequence, 2‐way crossover study in 13 healthy participants. On Day 1, 25 mg amitriptyline and 50 mg tramadol were orally administered concurrently, followed by a wash‐out period of at least 7 days. On Day 8, 30 mg of oral CBD oil was administered 1 h prior to amitriptyline and tramadol. On Days 1 and 8, participants presented to the clinical research unit in the morning and were discharged in the evening, briefly returning in the morning of Days 2 and 9 for pharmacokinetic sampling.

The study was conducted at the Centre for Human Drug Research in Leiden, the Netherlands. The study was authorized under the Clinical Trial Regulation (536/2014) by the Medical Ethics committee of Stichting Beoordeling Ethiek Biomedisch Onderzoek (Assen, the Netherlands) and was conducted according to the Dutch act on Medical Research Involving Human Participants (WMO) and in compliance with all International Conference on Harmonization Good Clinical Practice (ICH‐GCP) guidelines and the Declaration of Helsinki. This study was submitted via the Clinical Trials Information System (CTIS) and therefore registered prospectively on the corresponding public portal, under EU trial number 2023‐508428‐36‐00. This research has been funded by the Dutch Organization for knowledge and innovation in health, healthcare and well‐being (ZonMw), grant number 848120001.

Each participant provided written informed consent before any screening procedures were performed. Eligible participants included healthy male and female volunteers aged 18 to 45 years, with a body mass index of 18 to 32 kg/m^2^ and a minimum weight of 50 kg. The participants underwent a full medical screening, including medical history, a physical examination, blood chemistry and haematology, urinalysis and an electrocardiogram (ECG) to assess eligibility. Participants with a history of clinically significant epileptic disorders or traumatic brain injury were excluded, as tramadol and amitriptyline are known to lower the seizure threshold.^26^ Any participant who was a regular user of any illicit drugs (including cannabis), had a history of drug abuse or a positive urine drug test at screening, was excluded. Urine drug tests were repeated pre‐dose on Days 1 and 8.

A CYP genotyping sample was taken at screening and analysed by the Department of Clinical Pharmacy and Toxicology, Leiden University Medical Centre (Leiden, The Netherlands). The list of tested CYP enzyme variants is provided in the **Supplementary** Materials and phenotyping into poor, intermediate, extensive and ultra‐rapid metabolizers was performed according to the guidelines of the Dutch Pharmacogenetics Working Group.^27^, ^28^ Participants with a poor metabolizer phenotype for CYP2D6 were excluded, as their significantly reduced bioavailability of O‐desmethyltramadol,^29^ would hinder the assessment of the effect of cannabidiol on its metabolism. Furthermore, participants with the CYP2D6 ultrarapid metabolizer phenotype were also excluded, as this phenotype increases the metabolism of amitriptyline and its metabolite nortriptyline into their hydroxy metabolites, leading to low drug concentrations (and suboptimal therapeutic efficacy) in the clinical setting.^19^ Therefore, the CYP2D6 ultrarapid metabolizer phenotype was expected to hinder the assessment of cannabidiol effects on CYP2C19, CYP2C9 and CYP3A4‐mediated metabolism of amitriptyline to nortriptyline. Also, individuals with a poor metabolizer phenotype for CYP2C19 and CYP3A4 were excluded, as the low inherent enzyme activity in such individuals would hinder the assessment of inhibitory effects of cannabidiol.

No prescription and OTC medications were permitted within 14 days prior to dosing and during the course of the study, with the exception of paracetamol (up to 4 g/day) and ibuprofen (up to 1 g/day). No vitamin, mineral, herbal and dietary supplements were permitted within 7 days prior to dosing. Participants abstained from nicotine, alcohol and caffeine starting at 24 h prior to dosing and until the last PK sample of the visit. Any nutrients known to modulate CYP enzyme activity (e.g., grapefruit or Seville orange‐containing products or quinine‐containing drinks such as tonic water or bitter lemon) were not permitted from 3 days before the first visit until the end of the study. Participants were required to fast for at least 8 h overnight before dosing and for 3 h post‐dose.

All females of childbearing potential and all males were required to practice effective contraception during the study and to continue contraception for at least 90 days after the last dosing. Urine pregnancy testing was conducted in female participants on each study day prior to dosing.

### 2.2 Study drugs

The cannabidiol formulation used in this study was Clinican®, an almond oil‐based formulation containing 10% cannabidiol and manufactured by Clinical Cannabis Care (Breukelen, the Netherlands) and administered orally with 240 mL of water following an overnight fast. Single doses of 30 mg CBD were administered. Although CYP‐enzyme inhibition was expected to increase with multiple administrations of CBD, owing to accumulation and the time‐dependency of the inhibition, single administrations of CBD were deemed sufficient for this study, based on observed PK interactions induced by single doses of CBD in our previous research.^25^ Furthermore, we estimated that the 30 mg CBD dose is at the upper end of the range of CBD intake of patients who use CBD‐rich varieties of medicinal cannabis (or formulations containing THC and CBD, like Sativex®) for treatment of neuropathic pain symptoms,^23^ and as well as an approximately median dose for OTC CBD users. Cannabidiol was administered one hour prior to amitriptyline and tramadol (during visit 2 only) to offset the slower absorption expected from an oil‐based cannabinoid formulation, compared to the victim drugs.

Generic oral formulations of tramadol (50 mg tramadol hydrochloride capsule) and amitriptyline (25 mg hydrochloride tablet) were used in this study. These doses were at the low end of the therapeutic range, allowing for a safety margin for possible increases in plasma concentrations when co‐administered with CBD. Tramadol and amitriptyline were not known to induce or inhibit CYP‐enzymes in general, and no specific pharmacokinetic interactions between the two victim drugs were known to the investigators. Co‐administration of tramadol and amitriptyline was hypothesized to increase the risk of serotonergic syndrome and seizures, as both drugs have a monoaminergic effect and reduce the seizure threshold. However, this risk was deemed clinically irrelevant when low, single doses were administered to healthy volunteers. Based on these consideration regarding pharmacokinetics and safety, the two victim drugs were administered together in a cocktail design.

A wash‐out period of at least 7 days was considered sufficient, as it spanned over 5 terminal half‐lives of tramadol (~5–6 h),^30^ O‐DSMT (~7 h),^30^ amitriptyline (~25 h)^31^ and nortriptyline (~26 h).^32^

### 2.3 Pharmacokinetic assessments

Sampling for pharmacokinetics was performed pre‐dose and at 1, 2, 3, 4, 5, 6, 8, 12 and 24 h following the administration of amitriptyline and tramadol. Approximately 4 mL blood was collected via an i.v. catheter placed in an antecubital vein in the arm in polypropylene K2EDTA tubes. Following collection, samples were immediately cooled on ice. Within 30 min of collection, plasma was separated by centrifugation at approximately 2000 g for 10 min and promptly transferred into amber glass vials and stored at −70 to 80 °C. The samples were analysed by the iC42 Laboratory (Department of Anesthesiology, University of Colorado, Aurora, Colorado, USA). CBD and CBD metabolites were analysed using a validated assay that was previously published (CBD metabolite concentrations not reported in this manuscript).^33^ Concentrations of amitriptyline, nortriptyline, tramadol and O‐desmethyltramadol were determined using a validated assay that was based on a previously published high‐performance liquid chromatography tandem mass spectrometry (HPLC‐MS/MS) method.^34^ Details regarding the bioanalytical assays used to measure CBD, amitriptyline, nortriptyline, tramadol and O‐desmethyltramadol are located in the **Supplementary** Materials section.

R 3.6.1 for Windows (R Foundation for Statistical Computing/R development Core Team, Vienna, Austria, 2019), PKNCA package version 0.9.5, was used to calculate pharmacokinetic parameters. PK parameter calculations were based on actual sampling time. When an actual sampling time differed from the protocol time by more than 10% and at least 5 min, the concentration was excluded from the descriptive statistics, but not from the non‐compartmental analysis. AUC was calculated using the linear‐log trapezoidal rule. For the calculation of PK parameters, concentrations below the limit of quantification (BLQ) that occurred prior to the first quantifiable concentrations were considered as zero. BLQ concentrations that occurred between the first and last non‐BLQ values or occurred after the last non‐BLQ value were dropped (treated as ‘missing’). Metabolite/parent ratios were calculated based on the AUC_last_ and corrected for the molecular weight ratio.

### 2.4 Safety assessments

Safety and tolerability were assessed by monitoring of adverse events (AE) and vital signs.

### 2.5 Sample size

A sample size calculation was performed based on the AUCs of O‐desmethyltramadol and amitriptyline, as these parameters were expected to be most impacted by CBD. We expected the co‐administration of CBD to result in an approximately 50% increase in AUC of O‐desmethyltramadol and amitriptyline, based on our previous report.^25^ The sample calculation for a crossover study design required intra‐individual variability of the AUC of O‐desmethyltramadol and amitriptyline to be known; however, such data were not publicly available. Therefore, the intra‐individual variability was assumed to be (at most) equal to the known inter‐individual variability (which was a conservative estimate, as inter‐individual variability typically exceeds intra‐individual variability), and the calculated power is likely to be an underestimation.

For the sample size calculation for O‐desmethyltramadol, the AUC and variability reported in a previous trial with 25 participants dosed with 100 mg tramadol were used, in which a mean (SD) AUC of 669 (181) ng*h/mL was reported for O‐desmethyltramadol.^35^ Although the tramadol dose in this trial differed from the intended dose in our study, the estimate of the variability derived from this trial was deemed appropriate for the purpose of this sample size calculation. We calculated a sample size of 12 to have a power of 0.998 to detect a difference in means of 350 ng*h/mL (corresponding to a difference of 52%), assuming a standard deviation of differences of 225 ng*h/mL, using a paired t‐test with a 0.05 two‐sided significance level, in a crossover study design.

For the sample size calculation for amitriptyline, the AUC and variability reported in a previous trial with 28 participants dosed with 10 mg of amitriptyline were used, in which a mean (SD) AUC of 156 (87.8) ng*h/mL was reported for amitriptyline.^36^ Although the amitriptyline dose in this trial differed from the intended dose in our study, the estimate of the variability derived from this trial was deemed appropriate for the purpose of this sample size calculation. We calculated a sample size of 12 to have a power of 0.764 to detect a difference in means of 85 ng*h/mL (corresponding to a difference of 54%), assuming a standard deviation of differences of 100 ng*h/mL, using a paired t‐test with a 0.05 two‐sided significance level, in a crossover study design.

In addition to the statistical considerations provided above, the sample size of 12 participants was expected to be sufficient for a two‐way crossover drug–drug interaction study of CBD as perpetrator drug, based on previously published literature on the design of drug–drug interaction studies.^16^, ^37^

### 2.6 Statistical analysis

To establish whether significant treatment effects could be detected, the PK parameters were compared with a mixed effects model analysis of variance (ANOVA) with treatment as a fixed factor and participants as a random factor on log‐transformed data. The general treatment effect and specific contrasts were reported with the estimated difference and the 95% confidence interval, the least square mean estimates and the p‐value. All calculations were performed using SAS for Windows V9.4 (SAS Institute, Inc., Cary, NC, USA).

## 3 RESULTS

**FIGURE 2:**
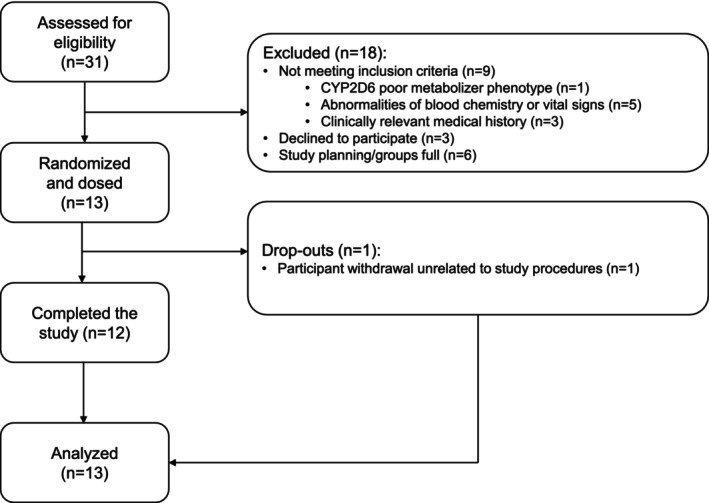
CONSORT study flowchart.

The clinical phase of the trial ran from December 2023 to March 2024. A total of 31 participants were screened, of which 13 (5 males/8 females) were enrolled in the study and dosed. Table 1 contains a summary of the baseline demographics. Of the 13 dosed participants, 1 (male) withdrew from the study following the first visit due to personal reasons and was replaced; 12 participants successfully completed the trial per protocol. Further details on participant enrolment are contained in the study flow diagram (Figure 2). All participants included in the study had either the extensive metabolizer or the intermediate metabolizer phenotype for CYP2C19, CYP2D6 and CYP3A4 (Table 1).

**TABLE 1:** Baseline participant demographics and CYP‐enzyme phenotypes.

	All participants (N = 13)
**Age (years)**	
Mean (SD)	24.4 (4.4)
Median (Min, Max)	22 (21, 35)
**Height (cm)**	
Mean (SD)	177.4 (9.7)
Median (Min, Max)	174.8 (164.5, 194.4)
**Weight (kg)**	
Mean (SD)	69.02 (8.17)
Median (Min, Max)	68.05 (58.95, 87.85)
**BMI (kg/m** ^**2**^ **)**	
Mean (SD)	22.0 (2.3)
Median (Min, Max)	21.3 (18.6, 25.8)
**Gender**	
Female	8 (61.5%)
Male	5 (38.5%)
**Race**	
Asian	1 (7.7%)
White	12 (92.3%)
**CYP2C19 phenotype**	
EM	10 (76.9%)
IM	3 (23.1%)
**CYP2D6 phenotype**	
EM	10 (76.9%)
IM	3 (23.1%)
**CYP3A4 phenotype**	
EM	12 (92.3%)
IM	1 (7.7%)

Abbreviations: BMI, body mass index; EM, extensive metabolizer; IM, intermediate metabolizer; max, maximum; min, minimum; N, number; SD, standard deviation.

Baseline concentrations of all analytes were below the limit of quantification (BLQ) for all participants, except for one participant with a baseline amitriptyline concentration of 0.85 ng/ml (lower limit of quantification: 0.75 ng/ml) during visit 2, which was treated as BLQ in the analysis. Number and percentage of BLQ samples per analyte are provided in the Supplementary Materials.

**FIGURE 3:**
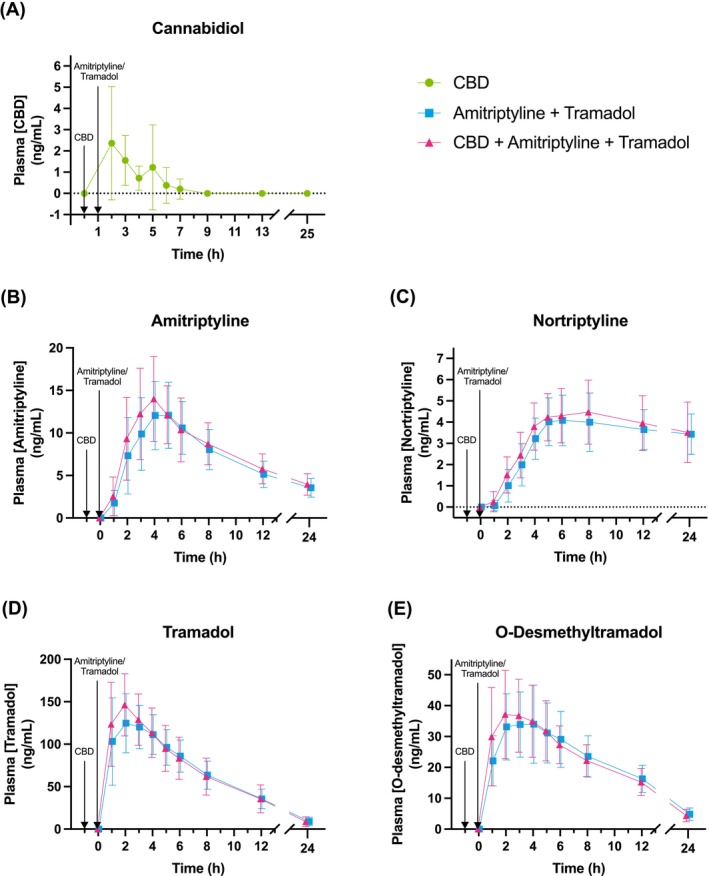
Concentration‐time profiles of a) cannabidiol, b) amitriptyline, c) nortriptyline, d) tramadol and e) O‐desmethyltramadol following oral administration, displayed as means with standard deviations.

Mean pharmacokinetic parameters of CBD, amitriptyline, nortriptyline, tramadol and O‐DSMT are provided in Table 2, and their concentration‐time profiles are displayed in Figure 3. CBD significantly increased the AUC_0‐24h_ of amitriptyline (LSM ratio 1.13, 95% CI: 1.01, 1.26, **p = 0.033)**, as well as its C_max_ (LSM ratio 1.17, 95% CI: 1.01, 1.36, **p = 0.041**). Furthermore, amitriptyline C_last_ (LSM ratio 1.14, 95% CI: 0.99, 1.32, p = 0.058) and tramadol C_max_ (LSM ratio 1.10, 95% CI: 0.99, 1.22, p = 0.064) increased after treatment with CBD but did not reach statistical significance. (Table 3). CBD had no statistically significant effects on other pharmacokinetic parameters of amitriptyline, tramadol and their metabolites, including the metabolite to parent ratios (Table 3).

**TABLE 2:** Pharmacokinetic parameters of cannabidiol, amitriptyline, nortriptyline, tramadol and O‐desmethyltramadol.

	Parameter	Unit	N	Mean	Geometric mean	SD	CV (%)	Geometric CV (%)	Median	Min	Max
**Cannabidiol**											
**Visit 2**	AUC_last_	h*ng/mL	12	6.9	5.3	5.4	78.0	87.4	6.2	1.7	20.3
	C_max_	ng/mL	12	3.5	2.9	2.4	68.1	68.4	3.0	1.1	9.8
	C_last_	ng/mL	12	1.1	1.0	0.3	25.3	25.0	1.1	0.8	1.7
	t_lag_	h	12						0.0	0.0	4.0
	t_max_	h	12						2.0	2.0	5.0
**Amitriptyline**											
**Visit 1**	AUC_last_	h*ng/mL	13	143.9	138.6	39.4	27.3	30.2	142.7	79.3	199.1
	C_max_	ng/mL	13	12.9	12.3	3.9	30.3	33.6	12.8	6.1	20.7
	C_last_	ng/mL	13	3.6	3.4	1.1	30.6	31.3	3.3	2.0	5.5
	t_lag_	h	13						0.0	0.0	1.0
	t_max_	h	13						4.0	3.0	6.0
**Visit 2**	AUC_last_	h*ng/mL	12	160.3	153.0	50.1	31.3	33.0	152.9	88.2	224.4
	C_max_	ng/mL	12	14.8	14.0	4.8	32.4	37.1	13.6	6.5	21.4
	C_last_	ng/mL	12	4.0	3.8	1.3	31.8	32.3	3.6	2.3	6.2
	t_lag_	h	12						0.0	0.0	1.0
	t_max_	h	12						4.0	2.0	5.0
**Nortriptyline**											
**Visit 1**	AUC_last_	h*ng/mL	13	77.9	75.3	20.9	26.8	27.9	73.0	40.7	126.0
	C_max_	ng/mL	13	4.5	4.4	1.2	25.7	26.4	4.5	2.5	7.1
	C_last_	ng/mL	13	3.4	3.3	0.9	27.6	30.8	3.3	1.5	5.5
	t_lag_	h	13						1.0	0.0	3.0
	t_max_	h	13						6.0	4.0	12.0
	MPR	‐	13	0.62	0.57	0.24	38.9	44.8	0.60	0.24	1.06
**Visit 2**	AUC_last_	h*ng/mL	12	84.5	80.6	27.6	32.7	32.8	77.3	48.0	133.7
	C_max_	ng/mL	12	4.7	4.5	1.5	32.3	31.7	4.7	2.7	8.3
	C_last_	ng/mL	12	3.5	3.3	1.4	40.4	39.7	3.2	1.9	6.2
	t_lag_	h	12						1.0	0.0	2.0
	t_max_	h	12						6.0	4.0	12.0
	MPR	‐	12	0.60	0.56	0.24	39.8	44.4	0.53	0.23	0.99
**Tramadol**											
**Visit 1**	AUC_last_	h*ng/mL	13	1168.4	1130.0	305.4	26.1	27.8	1239.2	753.8	1553.9
	C_max_	ng/mL	13	140.6	136.9	33.9	24.1	24.8	134.8	89.4	205.9
	C_last_	ng/mL	13	8.9	7.4	5.0	56.5	74.0	8.4	2.1	16.7
	t_lag_	h	13						0.0	0.0	0.0
	t_max_	h	13						2.0	1.0	4.0
**Visit 2**	AUC_last_	h*ng/mL	12	1211.0	1152.7	392.7	32.4	34.0	1159.2	727.7	1773.7
	C_max_	ng/mL	12	153.3	150.1	31.4	20.5	22.6	154.9	89.6	195.4
	C_last_	ng/mL	12	8.7	6.8	5.7	65.4	9.6	8.0	1.9	17.4
	t_lag_	h	12						0.0	0.0	0.0
	t_max_	h	12						2.0	1.0	3.0
**O‐desmethyltramadol**											
**Visit 1**	AUC_last_	h*ng/mL	13	412.0	400.4	105.2	25.5	25.0	388.6	283.5	605.4
	C_max_	ng/mL	13	36.7	35.0	12.6	34.3	32.0	31.1	24.1	63.2
	C_last_	ng/mL	13	4.8	4.4	2.0	41.8	46.3	4.1	2.0	9.1
	t_lag_	h	13						0.0	0.0	0.0
	t_max_	h	13						3.0	1.0	5.0
	MPR	‐	13	0.40	0.37	0.15	37.7	34.9	0.33	0.26	0.74
**Visit 2**	AUC_last_	h*ng/mL	12	411.1	399.5	102.9	25.0	25.4	396.1	282.2	603.9
	C_max_	ng/mL	12	39.5	37.6	13.8	34.9	31.8	33.6	25.8	70.3
	C_last_	ng/mL	12	4.4	4.0	2.0	46.0	48.5	3.9	2.0	7.9
	t_lag_	h	12						0.0	0.0	0.0
	t_max_	h	12						2.5	1.0	4.0
	MPR	‐	12	0.39	0.37	0.14	36.0	34.4	0.32	0.27	0.64

Abbreviations: AUC_last_, area under the concentration‐time curve from time zero to time of last measurable concentration; CBD, cannabidiol; C_max_, maximum concentration; C_last_, last observed concentration; CV, coefficient of variation; Geo, geometric; max, maximum; min, minimum; MPR, metabolite to parent ratio; N, number; SD, standard deviation; t_lag_, absorption lag time; t_max_, time to attain C_max_.

**TABLE 3:** Overall treatment effects on pharmacokinetic parameters.

			Back transformed				
						95% CI	
Analyte	Parameter	P‐value	LSM Visit 2	LSM Visit 1	Ratio of LSMs	Lower	Upper
**Amitriptyline**	AUC_last_	**0.033**	**156.24**	**138.56**	**1.13**	**1.01**	**1.26**
	C_max_	**0.041**	**14.46**	**12.33**	**1.17**	**1.01**	**1.36**
	C_last_	0.058	3.89	3.40	1.14	0.99	1.32
**Nortriptyline**	AUC_last_	0.309	79.54	75.31	1.06	0.94	1.18
	C_max_	0.580	4.51	4.36	1.04	0.91	1.18
	C_last_	0.926	3.28	3.30	0.99	0.86	1.15
	MPR	0.294	0.54	0.57	0.93	0.82	1.07
**Tramadol**	AUC_last_	0.259	1175.77	1130.01	1.04	0.97	1.12
	C_max_	0.064	150.95	136.87	1.10	0.99	1.22
	C_last_	0.690	7.16	7.41	0.97	0.80	1.16
**O‐desmethyltramadol**	AUC_last_	0.830	396.86	400.41	0.99	0.91	1.08
	C_max_	0.261	36.84	34.99	1.05	0.96	1.16
	C_last_	0.206	4.04	4.43	0.91	0.79	1.06
	MPR	0.351	0.36	0.37	0.95	0.86	1.06

The figures in bold indicate statistical significance. Abbreviations: AUC_last_, area under the concentration‐time curve from time zero to time of last measurable concentration; C_max_, maximum concentration; CI, confidence interval; C_last_, last observed concentration; LSM, least square mean; MPR, metabolite to parent ratio.

No serious adverse events occurred during the study. All AEs were transient and mild. The most common AEs were fatigue, reported by 10 (76.9%) participants after treatment with amitriptyline and tramadol and 10 (83.3%) participants after treatment with CBD, amitriptyline and tramadol and headache, reported by 3 (21.3%) participants and 3 (25.0%) participants, respectively. Dizziness was reported more frequently after dosing with amitriptyline and tramadol (4 [30.8%] participants) than after dosing with CBD, amitriptyline and tramadol (1 [8.3%] participant). An overview of AEs is provided in the Supplementary Materials.

## 4 DISCUSSION

CBD is known to cause potentially toxic pharmacokinetic drug interactions with multiple CYP‐substrate drugs due to its CYP‐enzyme inhibiting properties. However, the potential of CBD to cause pharmacokinetic interactions with analgesics that are commonly used for the treatment of chronic neuropathic had not been previously evaluated. The aim of this study was to characterize the potential pharmacokinetic interaction between CBD and common analgesics amitriptyline and tramadol at a CBD dose that is expected to be representative of common use in patients with neuropathic pain and OTC CBD users. In line with our hypothesis, 30 mg CBD significantly increased the plasma concentrations of amitriptyline, indicative of CBD‐induced inhibition of CYP‐mediated metabolism. Furthermore, the C_max_ of tramadol also increased, although not statistically significantly. The magnitude of the observed increases was limited, and it is unclear from the current study whether it is clinically meaningful. The observed slight increases in amitriptyline and tramadol concentrations may not be noticed by a patient and would certainly not cause toxicity. Thus, at exposures achieved in this study, CBD is safe in patients who use amitriptyline or tramadol.

However, there are reasons to expect that some patients may reach CBD exposures far exceeding those achieved in this study. Firstly, repeated or chronic CBD dosing is likely to be prevalent in patient populations, whereas single doses were administered in this study. CBD has a long terminal half‐life of ~60 h, and moderate accumulation is known to occur after repeated dosing.^38^ Additionally, CBD is a time‐dependent CYP‐inhibitor,^8^, ^9^ meaning that a prolonged exposure from repeated dosing will produce more inhibition than single doses of CBD. Secondly, as CBD is a highly lipophilic compound,^39^ its absorption is strongly influenced by the prandial state of the individual. Therefore, patients who take CBD in a fed state will reach higher CBD concentrations for any given dose and thereby increase the risk and magnitude of interactions.

The size of the interaction observed in this study was smaller compared to our previous report, where 30 mg CBD increased the mean AUC of 11‐OH‐THC, main active metabolite of THC, another CYP2C19 substrate, by 71% when administered 30 min before THC.^25^ Metabolism of THC is not identical to the metabolism of amitriptyline and tramadol, and the discrepant findings in the two studies may reflect differences in the way these drugs interact with CBD. Importantly, substantially lower plasma CBD concentrations in the current study (mean±SD C_max_ 3.5 ± 2.4 ng/ml) compared to the previous study (mean±SD C_max_ of 5.6 ± 4.6 ng/ml) likely contributed to a smaller effect size. The higher CBD concentrations in the previous trial could be attributed to the semi‐standardized light breakfast was administered prior to dosing, whereas participants in the current study were dosed in a fasted condition. Additionally, it is possible that the difference in drug formulation played a role in absorption: an oil formulation was administered in this study *vs*. a tablet formulation in the previous study. The relative contribution of prandial status and formulation remains uncertain in the absence of pharmacokinetic data of both formulations administered under similar prandial conditions.

The strengths of this study included the use of a CBD dose that is relevant to patients with chronic neuropathic pain (as well as the general population), and the choice of two widely prescribed analgesics as victim drugs, which makes the results of this research relevant to a broad patient population. The open‐label, fixed‐sequence, two‐way crossover design was well‐suited to address the research question, and the sample size was adequate for the hypothesized effect size. Exclusion of individuals with poor metabolizer phenotypes for CYP2D6, CYP2C19 and CYP3A4, as well as CYP2D6 ultra‐rapid metabolizers, removed the variability that such phenotypes would have introduced to the outcomes, since CBD was a priori expected to have little to no effect on victim drug metabolism in participants with such phenotypes.

Limitations include a pharmacokinetic sampling schedule that lacked sampling for CBD concentrations at earlier timepoints than 2 h post‐dose, which limited the characterization of the absorption phase of CBD and is likely to have affected the estimates of its pharmacokinetic parameters. However, characterization of CBD PK was not a primary goal of this study. Moreover, sampling up to 24 h post‐dose proved too short for full characterization of the pharmacokinetic profile of amitriptyline and especially nortriptyline. For amitriptyline, the terminal elimination phase was not fully captured by the sampling schedule, and for nortriptyline, most of the elimination phase. The estimates of the C_max_ are unaffected, but the estimates of the AUC are limited by the duration of the sampling period and are certain to be underestimation of the AUC_inf_. This limited sampling schedule is unlikely to have influenced the conclusions of this study, however. Inhibition of CYP‐mediated metabolism should be apparent from altered concentrations of amitriptyline and nortriptyline within 24 h post‐dose, if at all present, as evidenced by previous research comparing the metabolism of amitriptyline in patients with different CYP2D6 and CYP2C19 genotypes.^40^ Therefore, it is highly unlikely that an extended sampling period would have revealed an interaction that was missed in the current study. A larger sample size may have allowed for the detection of significant changes in the amitriptyline C_last_ and tramadol C_max_.

The proven potential of CBD to cause CYP‐mediated drug interactions, as well as the high (and growing) prevalence of its use,^4^ warrant future clinical studies of CBD‐induced drug interactions, despite the modest interaction observed in this study. Future investigations should especially focus on the interaction between CBD and commonly used analgesics, anxiolytics, hypnotics and antidepressants. Because OTC CBD is often used for treatment of pain and anxiety and to improve sleep and mood,^4^ it is precisely the typical prescription drugs for these indications, which are the most exposed to interactions with CBD. Finally, the large potential of CBD to cause drug–drug interaction induces us to speculate that the putative efficacy of CBD as an analgesic, anxiolytic or hypnotic could be in part explained by CBD‐induced, CYP‐mediated drug–drug interactions with conventional drugs used concomitantly, as has also been hypothesized for CBD's anti‐epileptic properties. Speculative, but not implausible, are positive reinforcement loops, where CBD‐induced interactions noticeably increase the effects of concomitant drugs, which leads patients to escalate CBD intake. Uncovering such interactions has the potential to improve individual pharmacotherapy, as adjustments of the victim drug dose appear to be a more sound pharmacotherapeutic strategy than reliance on unpredictable CYP‐mediated interactions induced by an expensive drug with adverse effects of its own.

## CONFLICT OF INTEREST STATEMENT

LK is the owner of Verdient Science and Tomori Pharmacology. All other authors declared no competing interests for this work.

## PATIENT CONSENT STATEMENT

Each participant provided written informed consent before any screening procedures were performed.

## PERMISSION TO REPRODUCE MATERIAL FROM OTHER SOURCES

N/A

## CLINICAL TRIAL REGISTRATION

This study was submitted via the Clinical Trials Information System (CTIS) and therefore registered prospectively on the corresponding public portal, under EU trial number 2023–508428–36‐00.

## Supporting information


**Table S1.** Lower limits of quantification (LLOQ) per analyte.
**Table S2.** Multiple Reaction Monitoring (MRM) ion transitions for CBD, amitriptyline, tramadol, their metabolites and the internal standards. Quantifier ion transitions are shown in bold.
**Table S3.** Number and proportion of post‐dose values below the limit of quantification (BLQ) per analyte.
**Table S4.** Overview of adverse events.

## Data Availability

Data from this study is available upon reasonable request to the corresponding author.
