# Supplementary material for: Low‐dose cannabidiol increases plasma concentrations of amitriptyline: A clinical drug–drug interaction study
Source: Br J Clin Pharmacol. 2025 Dec 15;92(6):1595–606. doi: 10.1002/bcp.70415 (PMC13206195; doi:10.1002/bcp.70415)
Supplement: Supplementary file 1 — Table S1. Lower limits of quantification (LLOQ) per analyte. Table S2. Multiple Reaction Monitoring (MRM) ion transitions for CBD, amitriptyline, tramadol, their metabolites and the internal standards. Quantifier ion transitions are shown in bold. Table S3. Number and proportion of post‐dose values below the limit of quantification (BLQ) per analyte. Table S4. Overview of adverse events. [file BCP-92-1595-s001.docx]

**Supplementary Materials**

***Study Title:***

“Low-dose cannabidiol increases plasma concentrations of amitriptyline: a clinical drug-drug interaction study”

***Authors and affiliations:***

A.A. Gorbenko^1,2^, T.E. Post^1^, P.K. Strugala^1^, E.S. Klaassen^1^, L.E. Klumpers^3,4,5^, S.J. de Visser^6^, C. Sempio^7^, J. Klawitter^7^, J.A.A.C. Heuberger^1^, G.J. Groeneveld^1,2^

1: Centre for Human Drug Research, Leiden, The Netherlands

2: Leiden University Medical Centre, Leiden, The Netherlands

3: Verdient Science, LLC., Denver, CO, US

4: Tomori Pharmacology, Inc., Denver, CO, US

5: Larner College of Medicine, University of Vermont, Burlington, VT, US

6: Centre for Future Affordable and Sustainable Therapy development (FAST), the Hague, the Netherlands

7: Department of Anesthesiology, University of Colorado, Aurora, Colorado, USA.

***CYP-enzyme genotyping***

*Tested CYP2C19 variants:*

*2 (rs4244285), *3 (rs4986893), *4 (rs28399504), *5 (rs56337013), *6 (rs72552267), *7 (rs72558186), *8 (rs41291556), *17 (rs12248560)

*Tested CYP2D6 variants:*

*2 (rs16947, rs1135840), *3 (rs35742686), *4 (rs382097), *6 (rs5030655), *7 (rs5030867), *8 (rs5030865, rs16947, rs1135840), *9 (rs5030656), *10 (rs1065852, rs1135840), *11 (rs201377835, rs16947, rs1135840), *12 (rs5030862, rs16947, rs1135840), *14 (rs72549357, rs16947, rs1135840), *15 (rs72549357), *17 (rs28371706, rs16947, rs1135840), *18 (rs765776661), *19 (rs72549353, rs16947, rs1135840), *20 (rs72549354, rs16947, rs1135840), *29 (rs59421388, rs16947, rs1135840), *41 (rs28371725, rs16947, rs1135840), *5 (gene-deletion), duplications

*Tested CYP3A4 variants:*

*2 (rs55785340), *17 (rs4987161), *22 (rs35599367)

***Bioanalytical Assay for Pharmacokinetics***

*Sample Preparation*

Briefly, 200 µL of plasma were extracted using 800 µL of protein precipitation solution that consisted of 0.2 M zinc sulphate in water: methanol (30:70, v/v) containing the following internal standards: CBD-d9, 7-OH-CBD-d3, 7-CBD-COOH-d3, Amitriptyline-d3, Nortriptyline-d3 and Tramadol-C13-d3. The lower limits of quantification (LLOQ) are provided in Table S1. Samples were vortexed for 10 minutes and centrifuged for 10 minutes (16,000g at 4°C). The supernatant was split in two aliquots and transferred to HPLC vials for LC-MS/MS analysis.

*Cannabinoids analysis*

For cannabinoids analysis, 250 µL of the samples were injected into a 3.0 · 5.0 mm, 2.7 μm extraction column (Halo C8 column, Advanced Material Technology, Wilmington, DE, USA) to perform online extraction. Samples were washed with a mobile phase of 70% 0.1% formic acid in water and 30% 0.1% formic acid in methanol. After 1 minute, the samples were eluted in backflush mode onto a 150 · 3.0 mm RP-Amide, 2.7 μm analytical column (Ascentis Express RP-Amide, Supelco, Sigma-Aldrich, Saint Louis, MO, USA). The mobile phase consisted of 0.04% formic acid in HPLC grade water (solvent A) and acetonitrile: methanol: isopropanol mixture (3:1:1, v/v, solvent B) and the flow rate was set at 0.55 mL/min. The MS/MS run in the positive multiple reaction monitoring (MRM) mode and the ion transitions listed in Table S1 were acquired. Instrument was controlled using the AB SCIEX Analyst Software (version 1.7.3).

*Amitriptyline and Tramadol analysis*

For the analysis of amitriptyline, tramadol and their respective metabolites, 8 μL of the samples were injected onto a 100 · 3.0 mm Kinetex F5, 100 Å analytical column (Phenomenex, Torrance, CA, USA). The mobile phase consisted of 0.2% formic acid in HPLC grade water (solvent A) and acetonitrile (solvent B) and the flow rate was set at 1 mL/min. The MS/MS run in the positive multiple reaction monitoring (MRM) mode and the ion transitions listed in Table S2 were acquired. Instrument was controlled using the AB SCIEX Analyst Software (version 1.7.3).

*Data analysis*

After the analysis was completed, peaks were integrated and the results were printed. Concentrations of CBD, amitriptyline, tramadol and their metabolites were quantified based on the analyte/ internal standard ratios using 1/x weighted and linear/quadratic fitted calibration curves depending on the analyte. All calculations were carried out using the SCIEX OS Software (version 1.6.1, Sciex, Concord, ON, Canada).***Table S1. Lower limits of quantification (LLOQ) per analyte.***

| **Analyte** | **LLOQ (ng/mL)** |
| --- | --- |
| Cannabidiol | 0.75 |
| 6α-OH-CBD | 1.50 |
| 6β-OH-CBD | 1.50 |
| 7-OH-CBD | 1.50 |
| 7-CBD-COOH | 1.50 |
| CBD-Glucuronide | 1.50 |
| Delta-9-Tetrahydrocannabinol | 0.75 |
| 11-OH-THC | 0.75 |
| THC-COOH | 0.75 |
| THC-COOH-Glucuronide | 7.50 |
| THC-Glucuronide | 0.75 |
| Amitriptyline | 0.75 |
| Nortriptyline | 0.75 |
| Tramadol | 0.75 |
| O-Desmethyl-tramadol | 0.75 |

***Table S2. Multiple Reaction Monitoring (MRM) ion transitions for CBD, amitriptyline, tramadol, their metabolites and the internal standards. Quantifier ion transitions are shown in bold.***

| **Analyte** | **Parent Ion**  **[M+H]^+^** | **Product Ion**  **[F+H]^+^** |
| --- | --- | --- |
| Cannabidiol | **315.2** | **193.1** |
|  | 315.2 | 259.3 |
| 6α-OH-CBD | **313.2** | **193.0** |
|  | 313.2 | 133.2 |
| 7-OH-CBD | **313.2** | **193.0** |
|  | 313.2 | 133.2 |
| 7-CBD-COOH | **345.3** | **299.3** |
|  | 345.3 | 327.5 |
| CBD-glucuronide | **315.2** | **193.1** |
|  | 315.2 | 259.3 |
| Amitriptyline | **278.2** | **233.2** |
|  | 278.2 | 191.2 |
| Nortriptyline | **264.2** | **233.2** |
|  | 264.2 | 191.1 |
| Tramadol | **264.1** | **58.0** |
|  | 264.1 | 41.8 |
| O-Desmethyl-tramadol | **250.1** | **58.1** |
|  | 250.1 | 41.8 |
| CBD-d9 | **324.4** | **202.3** |
| 7-OH-CBD-d3 | **316.2** | **196.1** |
| 7-CBD-COOH-d3 | **348.4** | **302.7** |
| Amitriptyline-d3 | **281.3** | **233.2** |
| Nortriptyline-d3 | **267.4** | **233.2** |
| Tramdol-C13-d3 | **268.3** | **58.1** |

***Table S3. Number and proportion of post-dose values below the limit of quantification (BLQ) per analyte.***

|  | **Total** | | **Day 1** | | **Day 8** | |
| --- | --- | --- | --- | --- | --- | --- |
|  | **Samples (n)** | **n (%) BLQ** | **Samples (n)** | **n (%) BLQ** | **Samples (n)** | **n (%) BLQ** |
| Amitriptyline | 225 | 7 (3.1%) | 117 | 4 (3.4%) | 108 | 3 (2.8%) |
| Nortriptyline | 225 | 27 (12%) | 117 | 17 (14.5%) | 108 | 10 (9.3%) |
| Tramadol | 225 | - | 117 | - | 108 | - |
| O-DSMT | 225 | - | 117 | - | 108 | - |
| CBD | 108 | 71 (65.7%) | - | - | 108 | 71 (65.7%) |

***Table S4. Overview of adverse events.***

|  | **Amitriptyline + tramadol (n=13)** | | **CBD + amitriptyline + tramadol  (n=12)** | |
| --- | --- | --- | --- | --- |
| **System Organ Class/  Preferred Term** | **Events N** | **Participants N (%)** | **Events N** | **Participants N (%)** |
| ANY EVENTS | 23 | 12 (92.3) | 16 | 10 (83.3) |
|  |  |  |  |  |
| GENERAL DISORDERS AND ADMINISTRATION SITE CONDITIONS | 10 | 10 (76.9) | 10 | 10 (83.3) |
| Fatigue | 10 | 10 (76.9) | 10 | 10 (83.3) |
|  |  |  |  |  |
| INFECTIONS AND INFESTATIONS | 1 | 1 (7.7) | - | - |
| Tonsillitis | 1 | 1 (7.7) | - | - |
|  |  |  |  |  |
| METABOLISM AND NUTRITION DISORDERS | - | - | 1 | 1 (8.3) |
| Increased appetite | - | - | 1 | 1 (8.3) |
|  |  |  |  |  |
| NERVOUS SYSTEM DISORDERS | 12 | 7 (53.8) | 4 | 4 (33.3) |
| Dizziness | 5 | 4 (30.8) | 1 | 1 (8.3) |
| Headache | 4 | 3 (23.1) | 3 | 3 (25.0) |
| Presyncope | 1 | 1 (7.7) | - | - |
| Sedation | 1 | 1 (7.7) | - | - |
| Somnolence | 1 | 1 (7.7) | - | - |
|  |  |  |  |  |
| VASCULAR DISORDERS | - | - | 1 | 1 (8.3) |
| Hypotension | - | - | 1 | 1 (8.3) |
